# Complete Genome Sequence of *Bacillus thuringiensis* Serovar rongseni Reference Strain SCG04-02, a Strain Toxic to *Plutella xylostella*

**DOI:** 10.1128/genomeA.00691-17

**Published:** 2017-09-28

**Authors:** Yajuan Fu, Yan Wu, Yihui Yuan, Meiying Gao

**Affiliations:** a Wuhan Institute of Virology, Chinese Academy of Sciences, Wuhan, China; b University of Chinese Academy of Sciences, Huaibeizhen, Huairou, Beijing, China

## Abstract

*Bacillus thuringiensis* (Bt) is widely used to control agricultural and forestry pests, though there are only a few available complete genome sequences of the Bt reference strain. Here, we report the complete genome sequence of *B. thuringiensis* serovar rongseni reference strain SCG04-02, which is toxic to *Plutella xylostella*.

## GENOME ANNOUNCEMENT

*Bacillus thuringiensis* (Bt) is a Gram-positive, spore-forming pathogenic bacterium of insects and is widely used in producing biological pesticides against a variety of agricultural and forestry pests (1, 2). The Bt strain SCG04-02 is the reference strain of *B. thuringiensis* serovar rongseni (H serotype H56) and was isolated from soil collected from Chongqin, China (3). The strain forms parasporal crystals with morphologies of bipyramidal and prolate ellipses and shows low toxicity to the larvae of *Plutella xylostella* (4).

Genome sequencing of SCG04-02 was performed by using Illumina HiSeq2500 with a paired-end library (insert size, 500 bp) strategy with read lengths of 150 bp. A total of 20,590,086 reads were obtained by Illumina sequencing, and low-quality reads were filtered by Quake (5). The clean reads were *de novo* assembled by SPAdes 3.5.0 (6) into 51 contigs. The assembly was further improved by comparing it with the complete genome sequences of the other strains in this species, such as strain BMB171 (GenBank accession number NC_014171) (7). The gaps between contigs were filled by primer walking and Sanger sequencing to obtain the complete genome sequence. Genome annotation was performed by using the NCBI Prokaryotic Genome Annotation Pipeline (http://www.ncbi.nlm.nih.gov/genome/annotation_prok/), and insecticidal genes were predicted by using gene data collected from the insecticidal gene database (http://www.lifesci.sussex.ac.uk/home/Neil_Crickmore/Bt/) and performing a local BLAST search. tRNA and rRNA genes were identified by tRNAscan-SE-1.23 and RNAmmer 1.2, respectively.

The genome size of SCG04-02 is 5,878,235 bp, containing a circular chromosome and four circular plasmids. The chromosome, which is 436,019 bp in length, harbors 5,463 coding sequences, 45 rRNA genes, and 91 tRNA genes. The G+C content of the chromosome is 35.3%, which is similar to those of the other isolates in the *B. thuringiensis* species. The plasmids, containing a total of 480 coding sequences, are named PSCG5 (5,650 bp), PSCG11 (11,179 bp), PSCG61 (61,350 bp), and PSCG364 (364,037 bp). The G+C contents of the plasmids range from 31.9% to 35.5%. Two insecticidal genes, BJG91_01545 and BJG91_01585, are found in the largest plasmid, PSCG364, to encode two Cry7 proteins. The amino acids between these two proteins identify at 65%. The product of BJG91_01545 showed 100% amino acid sequence similarity to Cry7Ba1, which is toxic to *Plutella xylostella* (8). The product of BJG91_01585 exhibits an amino acid sequence that identifies with existing proteins from the Cry7 type of proteins ranging from 56% to 65%, with much lower similarities to other types of insecticidal crystal proteins. Based on the classification of the Cry protein (9), the product of BJG91_01585 might be classified as a novel Cry7M-type protein.

The genome sequencing of SCG04-02 genome is valuable for the discovery of novel insecticide-associated genes and for understanding the genetic diversity of Bt.

### Accession number(s).

The genome sequence of the *B. thuringiensis* strain SCG04-02 has been deposited in GenBank under accession numbers CP017573 to CP017577. The strain is available in the *Bacillus* Genetic Stock Center (http://www.bgsc.org/) under BGSCID 4BT1.
